# Influence of ACE I/D Polymorphism on Circulating Levels of Plasminogen Activator Inhibitor 1, D-Dimer, Ultrasensitive C-Reactive Protein and Transforming Growth Factor β1 in Patients Undergoing Hemodialysis

**DOI:** 10.1371/journal.pone.0150613

**Published:** 2016-03-29

**Authors:** Sara Santos de Carvalho, Ana Cristina Simões e Silva, Adriano de Paula Sabino, Fernanda Cristina Gontijo Evangelista, Karina Braga Gomes, Luci Maria SantAna Dusse, Danyelle Romana Alves Rios

**Affiliations:** 1 Campus Centro Oeste Dona Lindu, Universidade Federal de São João del-Rei, Divinópolis/MG – Brazil; 2 Department of Pediatrics, Interdisciplinary Laboratory of Medical Investigation, Faculty of Medicine – Universidade Federal de Minas Gerais, Belo Horizonte/MG – Brazil; 3 Department of Clinical and Toxicological Analysis, Faculty of Pharmacy - Universidade Federal de Minas Gerais, Belo Horizonte/MG – Brazil; Escola Paulista de Medicina, BRAZIL

## Abstract

**Background:**

There is substantial evidence that chronic renal and cardiovascular diseases are associated with coagulation disorders, endothelial dysfunction, inflammation and fibrosis. Angiotensin-Converting Enzyme Insertion/Deletion polymorphism (ACE I/D polymorphism) has also be linked to cardiovascular diseases. Therefore, this study aimed to compare plasma levels of ultrassensible C-reactive protein (usCRP), PAI-1, D-dimer and TGF-β1 in patients undergoing HD with different ACE I/D polymorphisms.

**Methods:**

The study was performed in 138 patients at ESRD under hemodialysis therapy for more than six months. The patients were divided into three groups according to the genotype. Genomic DNA was extracted from blood cells (leukocytes). ACE I/D polymorphism was investigated by single polymerase chain reaction (PCR). Plasma levels of D-dimer, PAI-1 and TGF-β1 were measured by enzyme-linked immunosorbent assay (ELISA), and the determination of plasma levels of usCRP was performed by immunonephelometry. Data were analyzed by the software SigmaStat 2.03.

**Results:**

Clinical characteristics were similar in patients with these three ACE I/D polymorphisms, except for interdialytic weight gain. I allele could be associated with higher interdialytic weight gain (P = 0.017). Patients genotyped as DD and as ID had significantly higher levels of PAI-1 than those with II genotype. Other laboratory parameters did not significantly differ among the three subgroups (P = 0.033). Despite not reaching statistical significance, plasma levels of usCRP were higher in patients carrying the D allele.

**Conclusion:**

ACE I/D polymorphisms could be associated with changes in the regulation of sodium, fibrinolytic system, and possibly, inflammation. Our data showed that high levels of PAI-1 are detected when D allele is present, whereas greater interdialytic gain is associated with the presence of I allele. However, further studies with different experimental designs are necessary to elucidate the mechanisms involved in these associations.

## Introduction

Patients at end-stage renal disease (ESRD) undergoing dialysis therapy have increased morbidity and mortality compared with general population and approximately 40% of deaths in this population have been attributable to cardiovascular causes [1, 2]. Diabetes, hypertension, anemia, inflammation, vascular calcification, oxidative stress and alterations of the Renin Angiotensin System (RAS) are risk factors for cardiovascular disease (CVD) in patients under hemodialysis (HD) [2–5].

The RAS plays a central role in the regulation of blood pressure, hydroelectrolyte homeostasis and renal haemodynamics [2,3]. RAS mediators and enzymes have been linked to the pathogenesis of renal and cardiovascular diseases and also hypertension [3–5]. It is known that Angiotensin-Converting Enzyme (ACE) is a key enzyme in the RAS (zinc metallopeptidase) that converts angiotensin I to angiotensin II (Ang II) [6–8]. Angiotensin-Converting enzyme Isertion/Deletion polymorphism (ACE I/D polymorphism) is located in the intron of *ACE* gene and consists of either an insertion (I) allele or a deletion (D) allele leading to three possible genotypes: II, ID and DD. Human *ACE* gene is located in chromosome 17q23, where an insertion/deletion (I/D, dbSNP rs4646994) in intron 16 has been identified [9–11]. This polymorphism has been implicated in the development and progression of diabetic nephropathy, although conflicting results have been reported [12–14]. Furthermore, D allele of ACE I/D polymorphism has also be linked to cardiovascular diseases [15,16].

There is substantial evidence that chronic renal and cardiovascular diseases are associated with coagulation disorders, endothelial dysfunction, inflammation and fibrosis [17, 18]. In this regard, several markers of the fibrinolytic system and inflammation, such as D-dimer and C-reactive protein (CRP) exert a role in the pathogenesis of renal and cardiovascular disorders [19–21]. Elevated serum levels of CRP have been widely considered an important risk factor for atherosclerosis and coronary heart disease [22]. D-dimer is the smallest fibrin degradation product. Its plasma levels reflect either increase blood coagulation activation or fibrinolytic action pathway [19]. Considering fibrogenic markers, the transforming growth factor β1 (TGF-β1) is a multifunctional cytokine and key driver of fibrosis. It acts as a regulator of cell proliferation and collagen formation in cardiovascular and renal diseases [23, 24]. Plasminogen activator inhibitor 1 (PAI-1), a potent regulator of fibrinolysis, has also been involved in several physiopathological processes. Depending on the disease, its overexpression or deficiency may trigger deleterious outcomes [25]. In addition, previous studies have shown interactions between ACE I/D polymorphism, RAS and fibrinolytic cascade [26,27].

In this context, it is reasonable to hypothesize that ACE I/D polymorphisms might be associated with differences in plasma levels of hemostatic and inflammatory parameters. Therefore, this study aimed to compare plasma levels of ultrassensible C-reactive protein (usCRP), PAI-1, D-dimer and TGF-β1 in patients undergoing HD with different ACE I/D polymorphisms.

## Patients and Methods

### Patients

This cross-sectional study was performed in patients at ESRD under hemodialysis therapy for more than six months. It was first made a detailed analysis of 344 medical records of patients undergoing hemodialysis in Hospital das Clínicas/Federal University of Minas Gerais (UFMG) and Instituto Mineiro de Nefrologia in Belo Horizonte/MG, Brazil. Among 344 patients, 138 were selected for study and 206 were excluded. Exclusion criteria were prescription of oral anticoagulation therapy, patients with acute or chronic hepatic disease, systemic lupus erythematosus, malignant cancer, vasculitis and acute infections, history of renal transplantation, HIV positive, pregnancy and oral contraceptive use in women.

After investigation of the ACE I/D polymorphism patients were divided into three groups according to the genotype: DD group (deletion/deletion ACE polymorphism); ID group (insertion/deletion ACE polymorphism); and II group (insertion/insertion ACE polymorphism).

Patients required regular hemodialysis sections for 3–4 hours three times a week. Blood flow was usually 300-450mL/min with a dialysate flow at a constant rate of 500mL/min. All HD patients were dialyzed using low-flux polysulphone membranes and high-flow polysulphone membranes with bicarbonate-buffered dialysate. All patients received regular doses (100 a 150UI/kg/weight) of standard heparin before hemodialysis.

The Research Ethics Committee (COEP) of the Federal University of Minas Gerais approved study protocol (ETIC 099/07). All patients gave signed an informed consent term to participate in the study. Study protocol did not interfere with any medical prescription and/or recommendation.

### Assays

Blood samples were drawn from hemodialysis vascular access before the beginning of dialysis session at the first session of the week. Blood samples were collected in 0.129 mol/L sodium citrate in 9:1 volume ratio and ethylene diamine tetraacetic acid 8% (EDTA 8%). Blood collected in citrate was centrifuged at 3,800 rpm for 20 min at 4°C to yield platelet poor plasma (PPP) and blood collected in EDTA was used for DNA extraction. Samples were aliquoted and stored at -70°C until analyzed. Plasma levels of D-dimer, PAI-1 and TGF-β1 were measured by enzyme-linked immunosorbent assay (ELISA) using commercially available kits from American Diagnostica-USA and R & D Systems-USA. The determination of plasma levels of ultrasensible CRP was performed by immunonephelometry, using the CardioPhase hsCRP (Dade Behring, Germany) kit, according to manufacturer's instructions.

### Investigation of ACE I/D polymorphism genotypes

For investigation of the ACE I/D polymorphism, genomic DNA was extracted from blood cells (leukocytes), using the Wizard purification system (Promega Inc., Madison, WI, USA), following the manufacturer instructions.

ACE I/D polymorphism was investigated by single polymerase chain reaction (PCR). Briefly, ACE was obtained through one separate reaction, using oligonucleotides. The PCR primers were 5’GCCCTGCAGGTGTCTGCAGCATGT3’ (forward primer) and 5’GGATGGCTCTCCCCGCCTTGTCTC3’ (reverse primer). Final volume of PCR reaction mixture was 20 μL that contained 1mM of each primer (Invitrogen, São Paulo, SP, Brazil), 1.5 mM MgCl2, 0.2 mM of each deoxynucleoside triphosphate—dNTp (GIBCO BRL, São Paulo, SP, Brazil), 2.5 μL of 10X PCR Buffer and 0.5U of Taq polymerase (Phoneutria, Belo Horizonte, MG, Brazil).

Amplification was performed in a PT100 PCR thermocycler (MJ Research, Waltham, Massachusetts, USA) with initial denaturation step at 94°C for 2 min followed by 40 cycles consisting of denaturation at 94°C for 10 seconds, annealing at 60°C for 30 seconds and extension at 72°C for 30 seconds.

**Fig 1:**
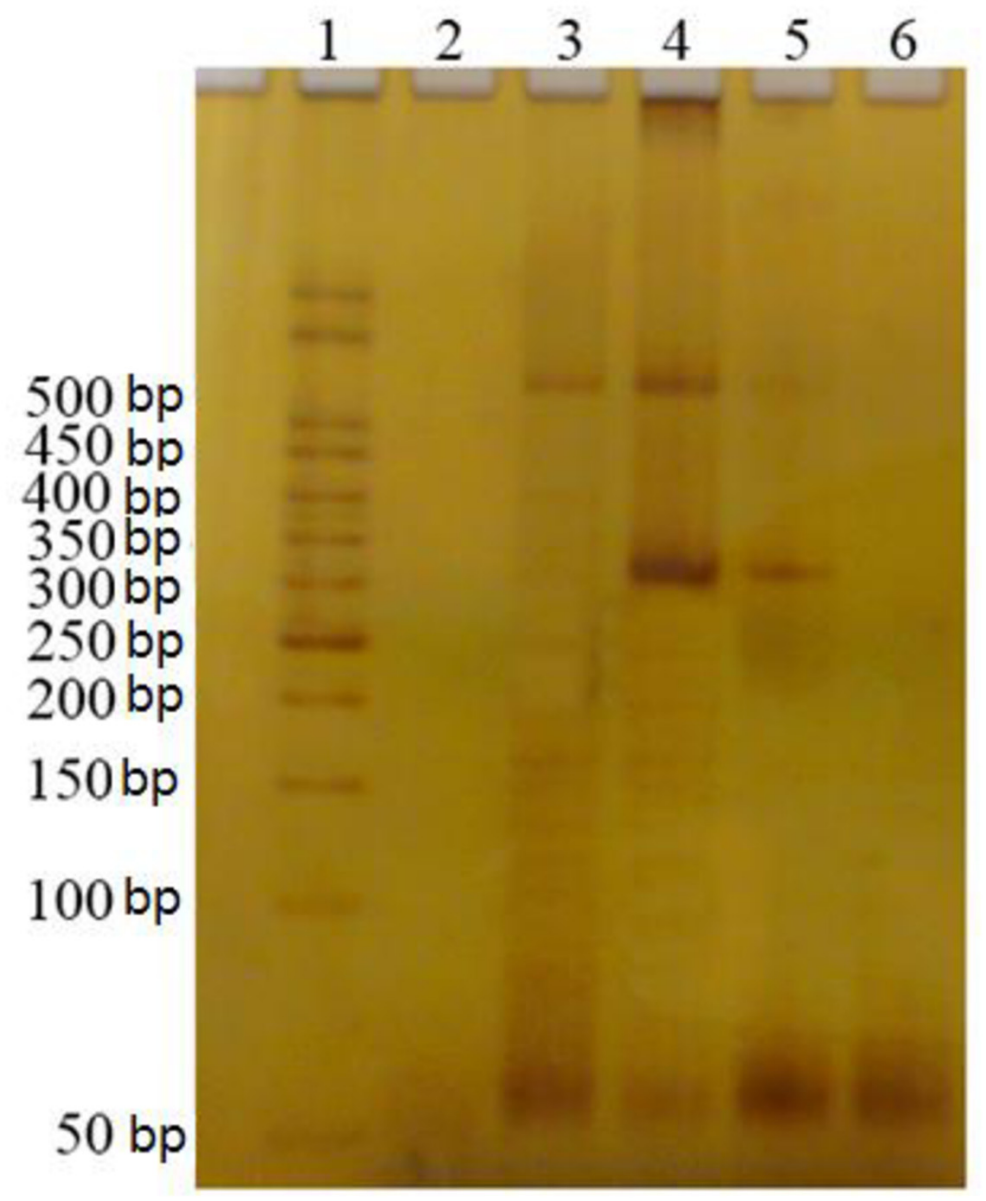
Electrophoresis on polyacrylamide gel stained with silver nitrate of PCR products to identify ACE polymorphism. Molecular weight standard of 50 bp; 3: DNA from individual homozygous II (599 bp); 4: DNA from individual heterozygous ID (599 and 312 bp); 5: DNA from individual homozygous DD (312 bp); 6: negative control.

PCR products were separated on polyacrylamide gel 8% and DNA was visualized by silver nitrate. This gel was prepared with 15 mL of polyacrylamide solution, 129 μL of ammonium persulfate and 15 μL TEMED (N, N, N ', N'-Tetramethylethylenediamine). DNA fragment sizes were 312 bp for the D allele and 599 bp for the I allele. Fragments displayed in the gels allowed to verify if the patient was heterozygous (ID: 599bp/312bp) or homozygous (II: 599bp and DD: 312bp). DNAs from previously typed individuals were included in each set of analyzed samples as control of enzyme activity (Fig 1).

### Statistical analysis

Data were analyzed by the software SigmaStat 2.03. Normality of variable distribution was checked using Kolmogorov-Smirnov test. Gaussian variables were reported as mean ± standard deviation (SD) and non-Gaussian variables as medians and interquartile range (IQ, percentile 25 and percentile 75). Analysis of variance (ANOVA) or Kruskal-Wallis test and Dunn post-test were used to compare differences between groups. p-values lower than 0.05 were considered statistically significant.

## Results

Among the total of 138 patients, 80 (58%) had DD genotype, 42 (30.4%) had ID genotype and 16 (11.6%) had II genotype. Clinical characteristics were similar in patients with these three genotype of ACE I/D polymorphisms, except for interdialytic weight gain (IDWG) (Table 1). Regarding the age, DD, ID and II patients had mean age of 50, 53 and 51 years, respectively. Most patients were males and mean body mass index (BMI) of the subgroups DD, ID and II were, respectively, 23.2, 24.1 and 23.2 kg/m^2^. In all subgroups, hypertensive nephrosclerosis was the main primary cause of chronic kidney disease (CKD).

**Table 1:** Clinical and epidemiological characteristics of studied patients in different genotype groups for ACE I/D polymorphism.

Parameters	DD group (n = 80)	ID group (n = 42)	II group (n = 16)	p value
Age (years)	50.0 (40–61.5)	53.0 (41.5–61.2)	51.0 (42–64.7)	0.854
Sex				
Male [n (%)]	41.0 (51.2)	22.0 (52.4)	10.0 (62.5)	0.710
Female [n(%)]	39.0 (48.8)	20.0 (47.6)	6.0 (37.5)	
BMI (Kg/m^2^)	23.2 (20.4–27)	24.1 (21.8–28.3)	23.2 (21.3–26.5)	0.417
Primary cause of CKD [n(%)]				
ypertensive nephrosclerosis	26.0 (32.5)	17.0 (40.5)	7.0 (43.7)	0.548
Glomerolupatias	25.0 (31.2)	8.0 (19)	2.0 (12.5)	0.153
Diabetic nephropathy	11.0 (13.7)	11.0 (26.2)	2.0 (12.5)	0.195
Polycystic kidneys	3.0 (3.7)	2.0 (4.8)	1.0 (6.3)	0.893
Other causes or etiology unknow	15.0 (18.7)	4.0 (25)	4.0 (8.3)	0.273
Pre-dialysis blood pressure				
SBP (mmHg)	140.0 (130–150)	135.0 (130–140)	130.0 (120–150)	0.463
DBP(mmHg)	80.0 (80–90)	80.0 (80–90)	80.0 (80–90)	0.697
HD time (months)	38.5 (16–96)	38.5 (18.7–91.2)	18.0 (13.5–69)	0.278
IDWG (g)	2890.4 ± 1095.6	3500.0 ± 1096.9	3325.0 ± 1344.9	0.017*
Diabetes [n (%)]	59.0 (73.7)	27.0 (65.8)	11.0 (68.7)	0.652
Medications [n(%)]				
ACE inhibitor	40 (50.0)	16 (38.1)	6 (37.5)	0.371
Angiotensin-receptor blocker	9 (11.3)	4 (9.5)	1 (6.3)	0.822
Diuretic	43 (53.8)	26 (61.9)	7 (43.8)	0.432
β-blockers	34 (42.5)	19 (45.2)	6 (37.5)	0.865
Calcium-channel blocker	34 (42.5)	21 (50)	6 (37.5)	0.619
Acetylsalicylic acid	16 (20.0)	11 (26.2)	5 (31.3)	0.535
Statins	13 (16.3)	8 (19.0)	3 (18.8)	0.917
Vitamin use	61 (76.3)	31 (73.8)	13 (81.3)	0.837
Insulin	13 (16.3)	11 (26.2)	2 (12.5)	0.324
Erythropoietin	69 (86.3)	37 (88.1)	11 (68.8)	0.159

*P< 0.05 DD x ID and DD x II. The normally distributed data were expressed as mean ± SD (ANOVA). The non-Gaussian data were presented as median (range interquartile). (Kruskal Wallis test). Frequencies (%) were evaluated by χ^2^ test. DD group: deletion/deletion group. ID group: insertion/deletion group. II group: insertion/insertion group. BMI: Body mass index; CKD: Chronic kidney disease; SBP: Systolic blood pressure; DBP: Diastolic blood pressure; HD time: time in hemodialysis; IDWG: interdialytic weight gain; ACE: angiotensin converting enzyme.

**Fig 2:**
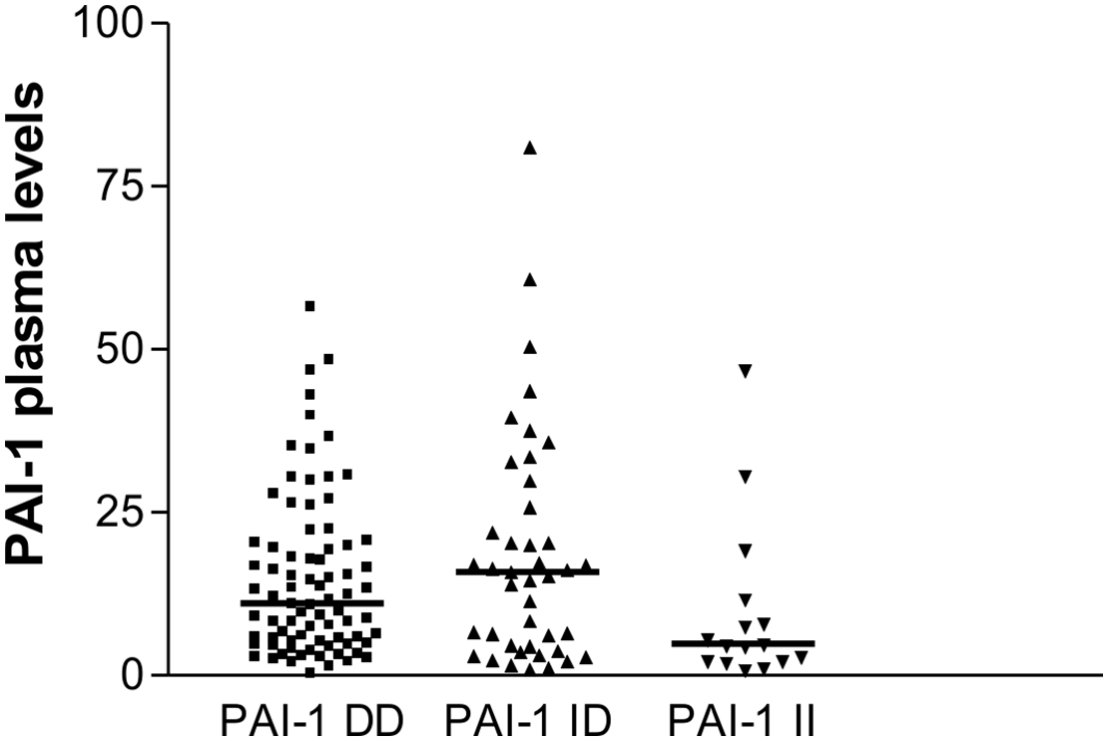
Distribution of PAI-1 (ng / mL) plasma levels in groups DD, ID and II. The horizontal lines indicate the medians obtained.

Laboratory parameters did not significantly differ among the three subgroups (Table 2), except for PAI-1 levels (Fig 2). Patients genotyped as DD (median of 12.1 ng/mL and IQ of 5.6–20.4 ng/mL) and as ID (median of 15.8 ng/mL and IQ of 4.6–27.1 ng/mL) had significantly higher levels of PAI-1 than those with II genotype (median 4.9 ng/mL and IQ of 2.4–10.9 ng/ml, p = 0.033, Fig 2). Despite not reaching statistical significance, usCRP levels were higher in patients carrying the D allele as shown in Table 2.

**Table 2:** Hemostatic and inflammatory parameters in different genotype groups for ACE I/D polymorphism.

Parameters	DD group (n = 80)	ID group (n = 42)	II group (n = 16)	P value
Erythrocytes x10^6^/mL	4.0 ± 0.6	4.0 ± 0.6	4.2 ± 0.7	0.464
Hemoglobin (g/dL)	12.5 (11.1–13.4)	12.1 (11–13.8)	13.0 (11.1–13.9)	0.776
Hematocrit (%)	37.5 (34–40.8)	36.7 (33.2–41.2)	39.1 (33.4–41.2)	0.677
Serum albumin	3.5 (3.3–3.7)	3.4 (3.3–3.6)	3.3 (3.1–3.8)	0.165
nPCR	1.1 (0.9–1.4)	1.1 (1.0–1.2)	0.9 (0.7–1.6)	0.503
PAI-1 (ng/mL)	12.1 (5.6–20.4)	15.8 (4.6–27.1)	4.9 (2.4–10.9)	0.033**
D-Di (ng/mL)	466.1 (250.2–813.8)	447.1 (278.7–1012)	387.3 (176.3–623.5)	0.420
TGF- β1 (pg/mL)	2740.6 (2195.6–3270.2)	2585.3 (2134.7–3395.6)	2544.6 (2149.8–3003)	0.633
usCRP (mg/L)	4.5 (2–9.6)	3.5 (1.6–9.7)	1.6 (0.7–5.3)	0.067

**P < 0.05 DD x II and ID x II. The normally distributed data were expressed as mean ± SD (ANOVA). The non-Gaussian data were presented as median (range interquartile). (Kruskal Wallis test). Frequencies (%) were evaluated by χ^2^ test. DD group: deletion/deletion group. ID group: insertion/deletion group. II group: insertion/insertion group. nPCR: normal protein catabolism rate; PAI-1: Plasminogen activator inhibitor type 1; D-Di: D-dimer; TGF-β1: Transforming growth factor-β1; usCPR: ultrasensitive C-reative protein.

## Discussion

We examined the association between ACE I/D polymorphism and clinical, hemostatic and inflammatory parameters in ESRD patients. Concerning clinical and epidemiological data, the only variable that differed according to ACE I/D polymorphism is IDWG. Our data shown that the presence of I allele is associated with higher IDWG, being 3,500g in ID and 3,325g in II versus 2,890g in patients with DD genotype. These findings may suggest an association between IDWG and I allele. IDWG, a measurement of HD compliance, is the result of salt and water intake between two dialysis sessions. In the literature there are two main causes that lead to the IDWG: nutritional status (calorie and protein intake) and water and salt intake [28]. In this study, in regard to the nutritional status, serum albumin and normal protein catabolism rate (nPCR) did not significantly differ among the three subgroups. Several studies have reported a relationship between ACE I/D polymorphism and salt sensitivity [29]. Salt sensitivity is a measure of how blood pressure responds to salt intake. Almost 50% of essential hypertension is salt sensivity, and this has been linked to an increased risk of cardiovascular events [30]. A positive association between I allele and salt sensitivity was previously found in essential hypertensive patients [31–33]. It means that the same amount of sodium intake produced a more pronounced increase in blood pressure in individuals with I allele. In our study we did not evaluate the relationship between ACE I/D polymorphism and salt sensitivity. However, we hypothetize that higher IDWG in patients who carry the allele I may be due to a higher sensitivity to the amount of sodium intake.

Our data showed that PAI-1 levels were significantly higher in patients carrying the D allele (DD and ID groups) compared to others without this allele (II group). According to our data, the same association between DD genotype and high PAI-1 levels was previously observed [34, 35]. We did not investigate the mechanisms beyond this association. However, one possible explanation is that our patients with D allele probably have higher levels and activity of ACE, as previously reported in other studies [11,36, 37]. Costerousse et al. showed that T-lymphocyte levels of ACE were significantly higher in healthy subjects with D/D ACE polymorphism than in subjects with other genotypes [38]. Indeed, the I/D polymorphism accounted for 47% of the total phenotypic variance of serum ACE, showing that the *ACE* gene locus is the major locus that determines serum ACE concentration [11]. In addition, several studies suggest a link between RAS and fibrinolytic system [39]. Ridker et al. and Yamamoto et al. [39, 40] proposed that high levels of ACE are associated with an increase in tissue conversion of angiotensin I (Ang I) into angiotensin II (Ang II). It was also observed that the DD genotype is associated with the enhanced conversion of Ang I to Ang II probably as a result of higher levels and activity of ACE [41]. Then, Ang II is enzymatically converted to angiotensin IV by aminopeptidases (localized on the endothelial surface), increasing the endothelial PAI-1 expression [39, 42, 43]. Lamentably, we were not able to confirm this hypothesis since ACE activity and/or Ang II levels were not measured in our patients.

ACE gene I/D polymorphism was studied frequently in cardiovascular diseases. Ishimitsu et al. [1] showed that D allele of the ACE gene polymorphism is an independent risk factor for cardiovascular diseases in long-term hemodialysis patients. Additionally, it has been shown that dialysis patients have an increase in PAI-1 levels, which becomes them vulnerable to cardiovascular risk [44, 25]. Guney et al. [45] showed that the ACE D allele and DD genotype were the major risk factors for coronary heart disease (CAD). Several studies have also revealed that ACE and PAI-1 play a role in CAD occurrence, since both molecules have a critical role in atherosclerosis and hypertension [46–48].

Previous studies emphasize RAS as a major contributor to hypertension [49, 50]. Besides it is associated with chronic inflammation in tissues and organs involved in key participants of blood pressure regulation, such as the kidneys and vessels [51]. Therefore, individuals who have the D would have more chances to develop hypertension and this increased pressure would lead to endothelial damage in parallel to the onset of inflammation. In this present study, we obtained a borderline P-value for usCRP levels increase (P = 0.067), and the highest levels were found in patients with the D allele compared to homozygous patients for the allele I.

CPR is produced by the liver under the control of various proinflamatory cytokines. usCRP is used as a marker of infection or activity parameter of ongoing inflammatory disease in ESRD patients [49]. It has been recognized that about 30–50% of predialysis and HD patients have serological evidence of an activated inflammatory response, which can be related to renal failure and may be a consequence of the dialysis process [50]. The bioincompatibility of dialysate, exposure of circulating mononuclear cells to the dialysis membrane, vascular access, may all be factors that enhance inflammation in HD patients [51]. Evidence from experimental and clinical studies has been accumulating that usCRP is not only a marker of inflammation but also contribute directly to the pathogenesis of atherosclerosis and its complications through a variety of mechanisms [52]. This led to the hypothesis of an accelerated atherogenesis in patients on dialysis. Moreover, CRP induces the synthesis and physiological functions of PAI-1 in the endothelium [53].

The RAS also has a direct influence on the progression of the atherosclerotic process via effects on endothelial function, inflammation, fibrinolytic balance, and plaque stability [54]. Therefore, the presence of D allele may be related to greater chance of developing atherosclerosis. We might speculate a relationship between ACE I/D polymorphism and PAI-1 levels in our study, since the levels of this marker are increased in patients who have the D allele (DD and ID groups) compared to patients without the D allele (II group). Thus, it is possible that patients who carrying the D allele have an increased chance of developing arteriosclerosis and other cardiovascular diseases compared to those without this allele.

In conclusion, this study suggests that ACE I/D polymorphisms could be associated with changes in the regulation of sodium, fibrinolytic system, and possibly, inflammation. Our data showed that high levels of PAI-1 are detected when D allele is present, whereas greater interdialytic gain is associated with the presence of I allele. However, further studies with different experimental designs are necessary to elucidate the mechanisms involved in these associations.
